# The Limiting Case of Amodal Completion: The Phenomenal Salience and the Role of Contrast Polarity

**DOI:** 10.3390/brainsci9060149

**Published:** 2019-06-24

**Authors:** Baingio Pinna, Livio Conti

**Affiliations:** 1 Department of Biomedical Science, University of Sassari, 07100 Sassari, Italy; 2 Faculty of Engineering, Uninettuno University, 00186 Roma, Italy; livio.conti@uninettunouniversity.net

**Keywords:** amodal completion, shape perception, perceptual organization, depth perception, visual illusions

## Abstract

In this work, we demonstrated unique and relevant visual properties imparted by contrast polarity in perceptual organization and in eliciting amodal completion, which is the vivid completion of a single continuous object of the visible parts of an occluded shape despite portions of its boundary contours not actually being seen. T-junction, good continuation, and closure are considered the main principles involved according to relevant explanations of amodal completion based on the simplicity–Prägnanz principle, Helmholtz’s likelihood, and Bayesian inference. The main interest of these approaches is to explain how the occluded object is completed, what is the amodal shape, and how contours of partially visible fragments are relatable behind an occluder. Different from these perspectives, amodal completion was considered here as a visual phenomenon and not as a process, i.e., the final outcome of perceptual processes and grouping principles. Therefore, the main question we addressed through our stimuli was “What is the role of shape formation and perceptual organization in inducing amodal completion?” To answer this question, novel stimuli, similar to limiting cases and *instantiae crucis*, were studied through Gestalt experimental phenomenology. The results demonstrated the domination of the contrast polarity against good continuation, T-junctions, and regularity. Moreover, the limiting conditions explored revealed a new kind of junction next to the T- and Y-junctions, respectively responsible for amodal completion and tessellation. We called them I-junctions. The results were theoretically discussed in relation to the previous approaches and in the light of the phenomenal salience imparted by contrast polarity.

## 1. Introduction

The presence of a multiplicity of objects within the natural environment, the loss of one spatial dimension during the projection of the image on the retina, and the inverse-optics problem reveal a true challenge that all visual systems must face and solve. This is the occlusion among objects within a three-dimensional space. Occlusion is indeed a complex issue to the computations of visual surfaces since many components, surfaces, and parts of objects cannot have any counterparts in a retinal image. Furthermore, most of the visual objects are projected on the retina only as fragments, pieces, or parts of something, which have to be computed and of which the full shape must be “completed” by neural mechanisms.

**Figure 1:**
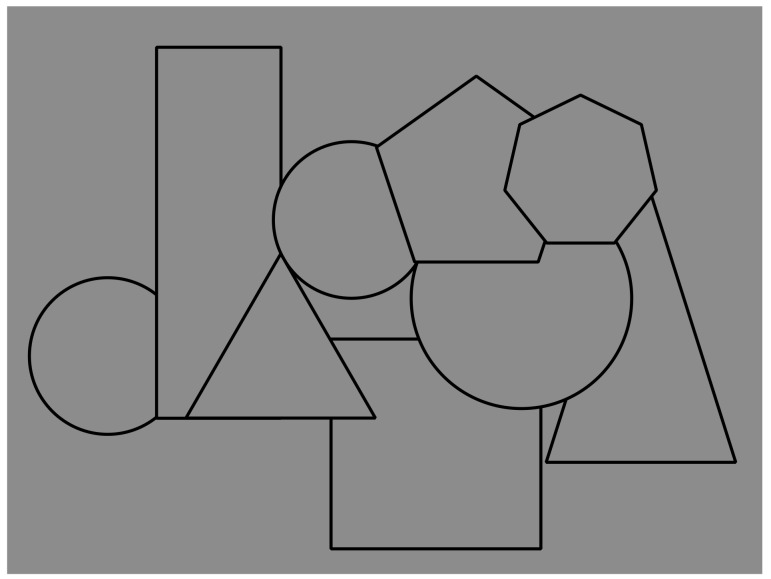
Amodal completion: the full and vivid completion of the visible portions of geometrical shapes behind other shapes.

**Figure 2:**
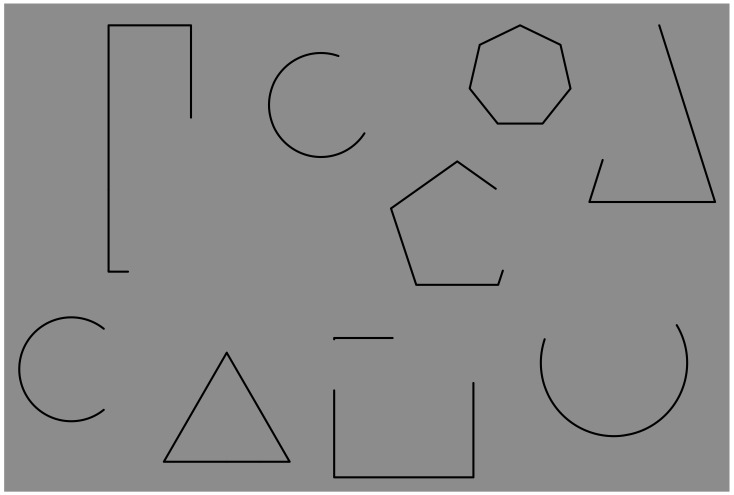
The visible portions of the amodal shapes perceived in Figure 1.

The occlusion and the resulting completion can be seen in Figure 1, where a bunch of overlapping geometrical shapes is visible. Phenomenally, three circles, one square, one rectangle, one triangle, one pentagon, and one heptagon are partially perceived due to their reciprocal occlusion. Only the heptagon and the triangle are visible in their full shapes. This simple and spontaneous description demonstrates the prompt and effortless full completion of most of the fragments actually illustrated in Figure 1 and more clearly shown separated in Figure 2.

The completion of visible portions of each shape as a single continuous object is what is known as “amodal completion”. Despite portions of boundary contours not actually being seen, the vivid outcome of a complete object unity is clearly perceived [1,2,3,4,5,6,7,8]. This is where the term “amodal” comes out. In summary, amodal completion is the intense sensory experience of completeness and unity of contours behind occluding objects (see also References [9,10,11,12,13]).

Amodal completion is likely the most common visual phenomenon and one of the most compelling problems of vision science. Psychophysical data demonstrated that several factors induce the perception of occlusion. They are mainly T-junction, asymmetry of Ts, good continuation, and closure [7,14,15,16,17,18,19,20]. Moreover, neurophysiological outcomes showed the role of neurons sensitive to amodal contours at higher visual levels [21,22,23,24]. Amodal completion is also related to processes of filling-in [7,25,26,27], thus showing that the visual world is not a mosaic of unconnected pieces of objects.

The outcomes, illustrated in Figure 1, are particularly suitable to be accounted for by Helmholtz’s likelihood principle [28] and Gregory’s ‘‘unconscious inference’’ [29,30]. They proposed that visual objects are similar to perceptual hypotheses postulated to explain the unlikely gaps within stimulus patterns according to what is perceived as the object that, under normal conditions, would be most likely to produce the sensory stimulation. In short, in Figure 1, partly occluded shapes are perceived because the other obvious possibilities, i.e., the fragments of Figure 2 abutting the occluding shapes, would require a coincidental and unlikely arrangement. Within the likelihood context, Rock [31,32] proposed the so-called avoidance-of-coincidences principle and claimed that the visual system tends to prevent interpretations elicited by coincidences. The visual system discharges coincidences tout court [33,34]. This is the case, for instance, of edges or junctions in one distal object that, through a specific view of a distal scene, accidentally coincide with edges or junctions in another distal object [33,35,36]. This principle can be proven selectively advantageous in the course of the phylogenetic development of the visual system.

This general idea of vision based on likelihood unconscious inference has been recently reconsidered in terms of probabilistic Bayesian inference [37,38,39,40,41,42,43]. It formally describes the optimal reasoning under uncertainty by specifying how to choose an outcome from a set of mutually exclusive hypotheses (Hs) on the basis of given stimulus patterns or data (D). According to Bayes’ rule, the posterior p(H|D), indicating how likely H is for a given D (i.e., the relative degree of resulting belief for each hypothesis), is the result of the convolution between the prior p(H), namely how likely H is in itself, and the conditional p(D|H), i.e., how likely D is under H (the likelihood function: how well D fits H). Therefore, Bayes’ theorem picks up the hypothesis that maximizes the posterior p(H|D).

In relation to Figure 1, the data are represented by the visual fragments projected on the retina and the hypotheses to be considered are the possible outcomes. Since the solution is undetermined, for example, due to the inverse-optics problem, Bayes’ theorem computes a probabilistic decision aimed at choosing the outcome that becomes the conscious perception according to the incoming stimulus pattern. This is supposed to occur by maximizing the posterior distribution. Therefore, the likelihood function, i.e., p(D|H), models aspects of optics and projection on the retina, while the prior, i.e., p(H), models the constraint and prior assumptions on the structure of the environment necessary to solve underdetermination.

In short, by reconsidering Figure 1 in terms of Bayes’ rule as stated in Helmholtzian likelihood principle and in the avoidance-of-coincidences principle, the most likely interpretation of Figure 1 is expected to be the set of full shapes previously described. This is also assumed to be the simplest solution.

Phenomenal simplicity refers to the notion that the visual system tends to extract a maximum of regularity. This idea is based on the simplicity–Prägnanz principle of Gestalt psychologists, who consider the visual system, like every physical system [44], as aimed at finding the simplest and the most stable organization consistent with the sensory inputs [45].

Simplicity and likelihood have been considered competing theories [31,46,47,48,49,50] that explain perceptual organization. The main difference is related to the fact that simplicity is based on a general principle of economy while likelihood is based on probability. In spite of this basic difference, they can be seen as two ways of considering the same visual process (see Reference [51]). As a matter of fact, the visual object that minimizes the description length is the same one that maximizes the likelihood. In other terms, the most likely hypothesis about the perceptual organization is also the outcome with the shortest description of the stimulus pattern.

Phenomenally, the most basic cues for amodal completion responsible for simplicity and likelihood are T-junctions [7,52,53,54]. The role of T-junctions within amodal completion can be explained by Occam interplay between priors and conditionals. Feldman [39] also suggested that T-junctions are mainly cues for segmentation since they, first of all, have to be segmented into visible parts of two different surfaces.

It should be pointed out that the most elementary condition of amodal completion is the figure–ground segregation, according to which a visual object partially occludes its back side and, at the same time, a portion of the background. Rubin [55,56] first studied figure–ground segregation as an essential process to the existence of visual objects by studying general principles of figure–ground segregation assumed as the atoms of a more general grammar of phenomenology of vision. Rubin’s main principles are surroundedness, size, orientation, contrast, closure, symmetry, proximity, convexity, and parallelism.

In Figure 1, the junctions among fragments are clearly related to their completion in object unities; however, not all of them are T-junctions. This is not an issue since all kinds of junctions can be reviewed in terms of the Gestalt principle of good continuation. For instance, T-junctions tend to be seen as visible parts of two different surfaces because the orientation of the vertical component of the “T” is dissimilar (orthogonal) from one of the two halves of the horizontal component, which is the best possible (good) continuation of the other half of the horizontal component. Both halves have the same orientation, the best or good possible continuation. This implies that T-junctions can be considered special conditions among many others, with intersecting contours not necessarily orthogonal of a Gestalt good continuation, that is *ipso facto* a generalization and a phenomenal explanation of the role of the junctions in eliciting amodal completion in Figure 1 [1,2,8,14,15,16,17,18]. Another kind of special junction is Y-junctions, eliciting juxtaposition and tessellation of surfaces (see next sections). In short, the bifurcation designed by the Y-junctions, in terms of good continuation, elicits two possible directions in equilibrium or equal probability. Therefore, the good continuation is not stopped by any boundaries as in T-junctions and is mainly responsible for the amodal continuation and completion. In this work, we proposed a new kind of junction, through limiting cases that we called I-junctions (see next sections). It is worth noting that T- and Y-junctions and, more generally, the good continuation can be read as a special case of similarity/dissimilarity among orientations of intersecting contours. This remark will be reconsidered below by matching this similarity due to orientations with the one elicited by contrast polarity.

**Figure 3:**
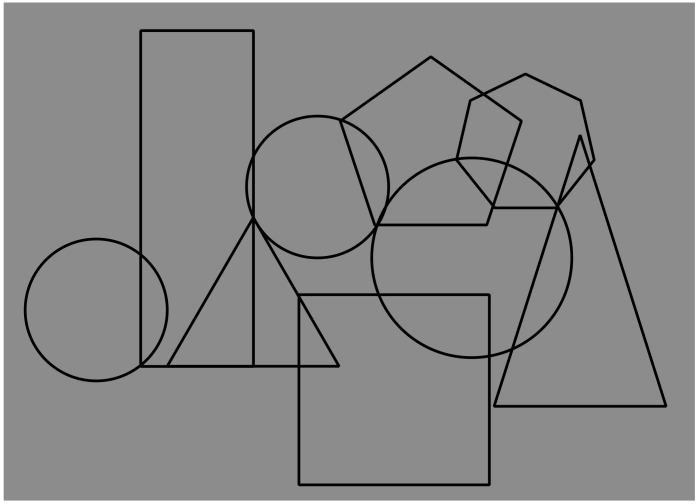
The invisible geometrical full and transparent overlapping shapes of Figure 1.

There is a further phenomenal attribute that is worth highlighting and that will be explored in the next sections. It is the amodal continuation. In short, it is the apparent and vivid outcome of continuation behind an occluding figure. As such, it is evidently related to amodal completion; nevertheless, it can be considered as operating more locally and partially independent from the more global-shaped organization due to amodal completion. For the time being, we direct reader’s attention to Figure 1, where the difference between amodal completion and continuation can be noticed. In Figure 1, the square underneath and the large triangle on the right appear distorted, respectively, in the square’s top-left corner and in the upper-corner of the triangle. These distortions or deformations are related to the continuation of the two fragmented sided of the figures that, continuing in their orientation, do not meet at the right point of shape regularity (for a more focused work on this matter, see Reference [7]). To better appreciate these effects, compare Figure 1 with Figure 3, where the complete, geometrical, full, and transparent overlapping of the inner shapes of Figure 1 is illustrated.

Given the segmentation induced by T-junctions, to perceive the intense sensory experience of completeness and unity of the amodal completion, it is necessary to perceive an occluding object and, thus, the perception of illusory depth. The continuous and smooth edges usually belong to the occluding object, whereas the intersecting (differently oriented) edges belong to the occluded object according to the unilateral belongingness of the boundaries [55,56] and the border ownership principle [7,28,57,58,59,60,61,62,63,64]. This is also the case of Figure 1, where the multiplicity of complete objects are perceived as placed at different depths and with some of them partially overlapping others.

This is also the case of the well-known Kanizsa’s triangle, where brightness enhancement and illusory contours are seen in the absence of a luminance or color change across the contour. Three black sectors and three angles, arranged respectively along the vertexes and sides of a virtual triangle, are perceived as three black disks and an outlined triangle in depth behind a triangle with clear boundary contours and brighter than the white background.

Based on Gestalt theory, Kanizsa [9,10] suggested that the necessary factor for the formation of the illusory figure is the presence of incompletenesses, or open figures, inducing amodal completion and closure processes that “create” complete perceptual elements behind a partially occluding illusory triangle.

Since amodal completion induces the formation of complete shapes and depth perception, the question that spontaneously attracted most scientists was “What is the role of amodal completion in shape formation?” [3,4,5,7,9,15,65,66,67].

The main target of this question is the shape completion of the partially occluded object, namely the shape that amodally completes the visible fragments. This question assumes the amodal completion as the cause of the formation of shape. Roughly speaking, the process of amodal completion is considered the main one responsible for the illusory depth and shape completion of the occluded object. Since the amodal shape is the object hypothesis of the perceived occluded object, what remains to be explained is the precise shape of the amodal objects. Simplicity and likelihood approaches are aimed at explaining how the occluded object is completed, its shape and information load, and how contours of partially visible fragments are relatable behind an occluder.

Complementary to this approach, there is another one that considers amodal completion not as the cause or the starting point but as the resulting effect, i.e., the end-point of the visual segmentation chain. The questions are, then, “What is the role of shape formation and perceptual organization in inducing amodal completion? Again, what are the perceptual conditions that elicit the segregation of occluded and occluding objects and, finally, amodal completion? Moreover, what is the role of the local contours, junctions, and termination attributes in eliciting the phenomenon of amodal completion?”

The answers to these questions allow us to understand the perception of illusory depth and the emergence of occluding and occluded objects. Within this perspective, amodal completion is reconsidered in terms of shape formation and, as such, reduced to elementary and more general principles of grouping and figure–ground segregation. Within the last question, amodal completion is considered as a visual phenomenon, while in the previous question (“What is the role of amodal completion in shape formation?”), it is assumed as a process aimed at explaining the perceived amodal shape.

On these bases, the main purpose of this work is to answer the last questions and to investigate more in detail amodal completion as a phenomenon not taken for granted but as the final outcome of perceptual organization: the result of upstream dynamics of shape formation and grouping principles. We assumed that this perspective could be a good candidate to test Helmholtz’s likelihood principle, the avoidance-of-coincidences principle, and Bayes’ framework.

The contrast polarity, with its related similarity/dissimilarity outcomes, is the main grouping and ungrouping attributes used in the next sections to explore amodal completion as a visual phenomenon.

**Figure 4:**
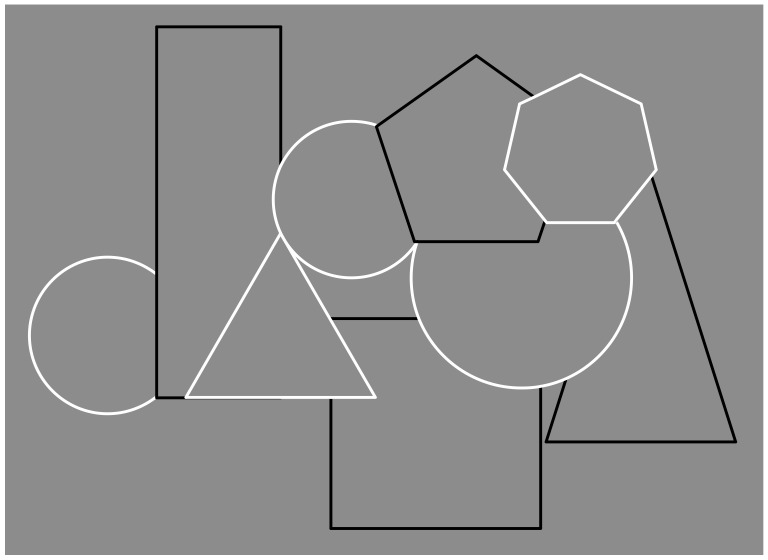
When the contrast polarity plays synergistically with other factors (T-junctions and good continuation), amodal completion and depth segregation are more salient than those perceived in Figure 1.

**Figure 5:**
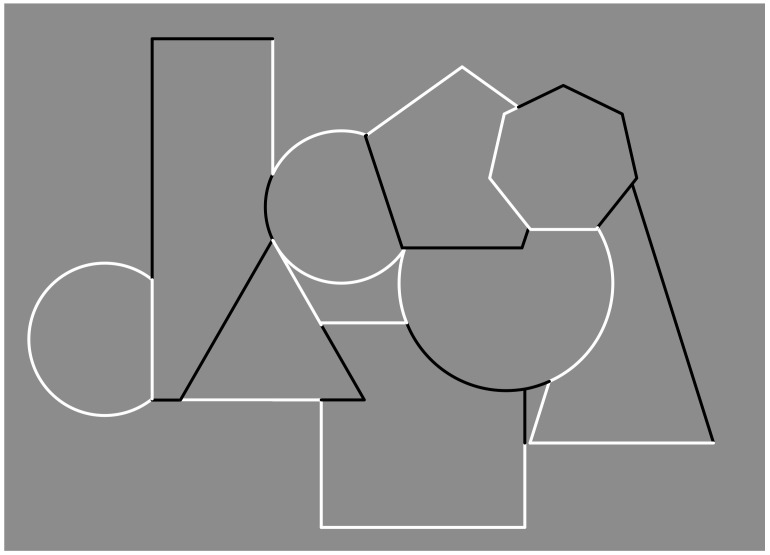
The results of Figure 4 are now partially and slightly disrupted, parceled, and camouflaged due to the contrast polarity pitted against T-junctions and good continuation.

Recently, contrast polarity has been demonstrated as an effective visual attribute in imparting strong grouping effects within a pattern of stimuli [68,69,70,71]. It can put together otherwise segregated elements or disrupt joined components. Figure 4 and Figure 5 show respectively the grouping and ungrouping properties induced by contrast polarity in favor or against the good continuation and T-junctions of Figure 1.

Without going into detail, in Figure 4, the salience of both depth segregation and amodal completion of partially occluded shapes are stronger than the ones illustrated in Figure 1. Under these conditions, contrast polarity plays synergistically with good continuation and T-junctions. On the contrary, in Figure 5, since the contrast polarity is now pitted against the same factors, both amodal completion and the unity of the outcomes of Figure 4 are partially disrupted, parceled, and camouflaged.

The new conditions studied in the next sections will deepen the role and strength of contrast polarity when it is pitted against or in favor of other grouping principles of perceptual organization.

## 2. Materials and Methods

### 2.1. Subjects

Different groups of 20 undergraduate students participated in each experiment described in the next sections. About 20% of the subjects had some basic knowledge of visual illusions, Gestalt psychology, and amodal completion, and the others were totally naive both to the stimuli presented here and to the purpose of the experiments. They were about 50% male and female, and all had normal or corrected-to-normal vision.

### 2.2. Stimuli

The stimuli were the figures, illustrated in the next sections, mainly based on a cross geometrically composed of five adjacent squares or a cross made up of two-centered and intersected orthogonal rectangles of equal size. Further stimuli were juxtaposed, overlapped, and isolated polygons. The stimuli were displayed on a 33-cm color CRT monitor (Sony GDM-F520 1600 × 1200 pixels, refresh rate 100 Hz, Sony, Tokyo, Japan) driven by a MacBook computer with an NVIDIA GeForce 8600 M GT in ambient illumination provided by a Osram Daylight fluorescent light (250 lx, 5600 K). The overall sizes of the figures were approximately 5 degrees. The luminance of the white elements was 122.3 cd m^−2^. Black components had a luminance value of 2.6 cd m^−2^. The gray background luminance was 62.5 cd m^−2^, about halfway between the white and black line components.

Viewing was binocular in the frontoparallel plane at a distance of 60 cm from the monitor.

### 2.3. Procedure

Given the complexity of the questions we are going to answer, we addressed related issues by showing novel and possibly fruitful conditions based, first of all, on self-evident perceptions and, secondly, on experimental phenomenology [58,59,71,72,73,74,75], including qualitative observations under controlled conditions rather than psychophysical results. Our purpose was to answer the questions by means of phenomenal outcomes that, being as strong as possible, could isolate the qualitative role of perceptual principles useful in testing the effectiveness of predictions and theories. Therefore, the stimuli have been designed to serve as limiting conditions or *instantiae crucis* (crucial instances) aimed at testing general theoretical statements through detailed phenomenal properties. Each of our experiments can be, therefore, read as an *experimentum crucis* (see also References [45,76,77,78]).

The procedure was twofold in line with the classical ones used by Gestalt psychologists (see also References [9,10,45,79,80,81,82,83,84]). The first is the phenomenological free-report method, through which naive subjects were asked to report anything they see in the following series of visual stimuli. The second is a more quantitative method, according to which subjects were instructed to rate (in percent) the descriptions obtained in the phenomenological experiments.

Phenomenological task—The task of the subjects was to report spontaneously what they perceived for each stimulus by giving, as much as possible, an exhaustive description of the main visual outcomes. The descriptions were judged by three students of linguistics that were totally naive to the hypotheses in order to get a fair representation of the responses of the subjects. All reports occurred fast and spontaneous. Observation time was unlimited, with the observers looking at the stimuli during their report.

Participants could make free comparisons, add comments as afterthought, and view the displays in different ways and from different distances. Subjects could receive suggestions/questions of any kind, such as “What is the shape of each component? What is the whole shape?” All the variations and possible comparisons occurring during the free exploration were noted by the experimenter. They could also match the stimulus with every other one they (or the experimenter) considered appropriate. This degree of freedom is in line with experimental phenomenology and aimed at more stable outcomes. The selection of stimuli could involve opposite conditions, controls, and possible comparisons between stimuli. The stimuli were presented randomly to minimize biases and past experience.

Scaling task—Subjects were asked to rate (as percent) the main descriptions resulting from the previous phenomenological task. At this stage, new groups of 20 subjects were asked to scale the relative strength or salience (in percent) of the main outcomes. Their task was literally: “please rate whether this statement (e.g., “five-squares juxtaposed and placed on the same depth plane” or “two-rectangles placed at a different depth”) is an accurate reflection of your perception of the stimulus on a scale from 100 (perfect agreement) to 0 (complete disagreement)”. We report below descriptions of which the mean ratings were greater than 85 across all experiments (about these procedures, see also References [58,59,68,82,85]). Critically, the statements rated were based on a careful analysis of previously obtained spontaneous descriptions, so the subjects were not being forced to rate appearances that no one had reported before.

In the following sections, the reported descriptions are incorporated within the text to aid the reader in the stream of argumentations.

## 3. Results

### 3.1. Contrast Polarity, Similarity, and Good Continuation

The starting question is “What is the role of shape formation and perceptual organization in inducing amodal completion?” To answer this question, the main purpose is studying the complexity of the perceptual organization through contrast polarity, which is useful to understand the complexity of amodal completion and to test the effectiveness of the theoretical approaches previously described. The role of contrast polarity will be investigated in relation to T-junctions, good continuation, and regularity.

**Figure 6:**
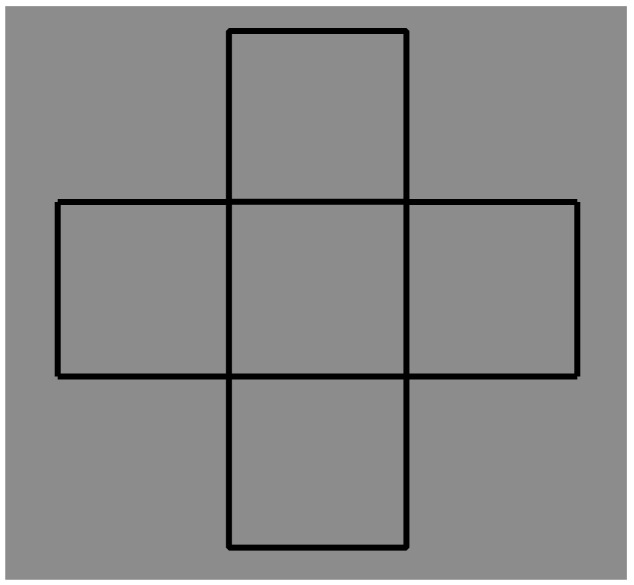
A cross geometrically composed of five adjacent squares or a cross made up of two-centered and intersected orthogonal rectangles of equal size?

The basic condition for the first set of stimuli is the Greek cross-like shape with all arms of equal length and equal sides illustrated in Figure 6. The cross is composed of five adjacent squares. This geometrical description becomes challenging for our purposes when it is compared with the following phenomenal report: a cross composed and made up of two centered and intersected orthogonal rectangles of equal size. The rectangles are overlapping, defined only in their boundaries or perimeter (outlined only) with the inner edges totally transparent or empty.

In terms of simplicity and Bayes’ inference, this description is privileged in relation to the cross composed of five juxtaposed squares. From the perspective of Bayes’ inference, the geometrical five-squares solution, although unlikely, is processed anyway.

The phenomenal outcome is privileged, primarily, because the number of elements is minimized: two rectangles vs. five squares. Secondly, the ambiguity related to the number of elements is also reduced. In this regard, we asked our subjects to answer the following questions, “Now, try to perceive adjacent squares; what is the number of squares?” Since we have already answered this question, namely “five”, the question is apparently trivial. Nevertheless, looking more carefully at the stimulus, the number of squares could be four or five. In fact, eleven out of twenty subjects answered “four”. This implies that they perceived the central square not as a filled surface but as an empty space. This result is mainly due to Rubin’s unilateral belongingness of the boundaries (border ownership). Indeed, to create the pattern of Figure 6, the juxtaposition of the four squares at the angles is sufficient. The central square can be implicit, that is, given by construction. Moreover, phenomenally, the boundaries (i.e., the four surrounding squares) are perceptually the best candidates to describe the whole shape (the cross). The nine remaining observers perceived five filled squares.

Actually, the geometrical and phenomenal perspectives can be considered as two different ways of watching the stimulus, that is locally or globally, and under these conditions, there is no ambiguity: respectively, five squares and two rectangles. Furthermore, the geometrical way reveals a phenomenal tessellation, while the phenomenal way highlights an overlapping and intersection outcome.

There is another more effective phenomenal quality favoring the two-rectangles solution. This is the good continuation principle that makes each longer contour appear as unique and seamless and as a unity. It prevents the longer side of the two orthogonal rectangles from being broken in three pieces and then brings about the adjacent-squares solution. The breakdown of the longer contours is the opposite of the tendency of the elements with more similar orientations to be grouped together. Actually, each intersection is a bifurcation and the visual system is expected to compute a Bayesian decision. In phenomenal terms, the result of Figure 6 states, “All else being equal, whenever there is a bifurcation and changes of direction, they can be prevented (minimized), unless otherwise stated, they do not change”. An algorithmic way to code the principle of good continuation could be “Avoid (or minimize) changes” or “Follow the minimum changes”. By writing “unless otherwise stated”, we are referring to the new conditions that are going to be introduced (see next figure).

There is a further complexity to be considered in the two-rectangles solution: (i) the perceived overlapping of the rectangular wires and (ii) which is in front and which is in the back. The perceived order of superimposition in Figure 6 is perceived as reversible, i.e., alternately, the horizontal and vertical rectangles can be perceived one in front of the other. This problem has been studied first by Petter [86], who discovered an interesting effect concerning conditions where a full black irregular pattern may be seen as made up of independent surfaces separated in depth and delineated by illusory contours in the area of apparent intersection and stratification. Petter also studied patterns formed by transparent surfaces or formed by outlined surfaces similar to the ones illustrated in Figure 6. He suggested that the perceived stratification occurs according to a general rule stating that the surface with the shorter contours, placed in the region where the surfaces look superimposed, has a greater probability of appearing in front of the other surface [9,87,88]. Since the length of the contours is equal, then the expected order of superimposition should be and it is reversible. Shipley and Kellman [87,88] also demonstrated that completion occurs not only for two-dimensional shapes but also for one-dimensional line figures. Moreover, inferred shapes can happen for curvilinear as well as linear elements.

Summing up, while the geometrical five-squares are seen as juxtaposed and all placed on the same depth plane, the two-rectangles are perceived as placed at a different depth. It seems that the minimization of the number of elements implies the emergence of depth. The reduction of the number of components implies the addition of a new dimension, useful to explain the reduction.

These preliminary descriptions of Figure 6 seem comprehensive and complete. However, they are just two among a plethora of possible results not easy to calculate, although, within Bayes’ inference, they are all taken into account and computed. Even with a simple stimulus like this, the computation could entail an exponential algorithmic complexity.

**Figure 7:**
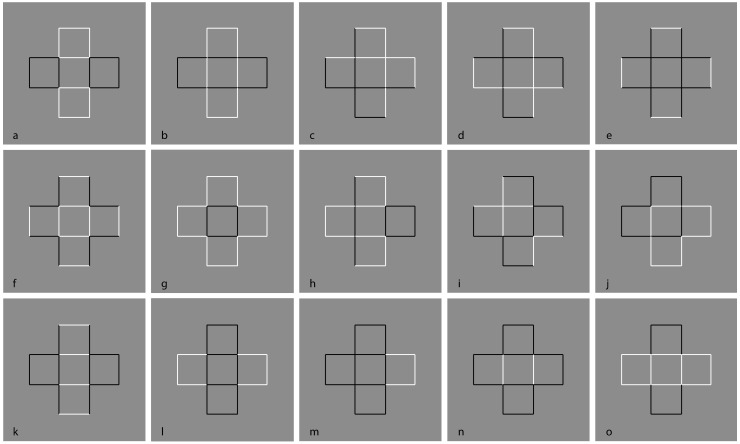
Conditions demonstrating the dominance of the contrast polarity over good continuation, T-junctions, and regularity.

Moreover, since the likelihood of all the combinatorial possibilities, except the two here considered, is about zero, then a fortiori it is an algorithmic waste to compute all the possible combinations. Given the fast and effortless answer of the subjects, it seems that the visual system knows exactly what to see without any need to compute combinations. This is due to the good continuation that preserves the visual system from computing futile and unlikely combinations. It is worthwhile to report that when subjects were asked to make an effort to perceive other possible combinations, they obstructed the task, stating that they are unable to calculate and perceive them. In Figure 7, some of these combinations are highlighted through the contrast polarity.

Another point that is worth being considered is related to the simplicity/Prägnanz of the previous results. More particularly, among the plethora of possible combinations, we can ask if there are other combinations that could be simpler than the two-rectangles outcome. Theoretically, the number two can be reduced to one if only the cross could be perceived. Actually, this is what has been reported, but phenomenally this answer is slightly but significantly different from a full perception of a cross: “a cross made up of two rectangles”. As a matter of fact, this is not a cross, i.e., a secondary and indirect or virtual object made up of two primary rectangles. The point remains open, but the answer might be possible among the phenomenal results of Figure 7.

If in Figure 6, T-junctions and good continuation are the main principles involved (closure should also be considered, but within this stimulus, they are balanced), in Figure 7 the similarity principle elicited by contrast polarity is now pitted in favor or against the previous results and the principles involved. More in detail, in Figure 7a, both T-junctions and good continuation are broken apart and the unique phenomenal outcome is four squares, two white and two black. The inner squared region is empty, as previously described, but with the difference that, in Figure 7a, this solution is much stronger than the one in Figure 6 and totally dominant against all the others, that are unlikely.

In Figure 7b, the overlapping two-rectangles solution is restored and made more salient by the contrast polarity. Under these conditions, the white rectangle has been reported as more vividly perceived popping out in front of the black one. In Figure 7c,d, the two rectangles are perceived with a clear bulging and volumetric effect. While in Figure 7c, both rectangles appear as convex, in Figure 7d the horizontal rectangle is seen as concave.

These volumetric effects can be explained on the basis of the assumption that the direction of the light source is from above. Actually, the qualitative volume interpretation of a visual object comes from the light-source constraint. If the light source lies in the concave side of a cast shadow contour of the rectangle, it should be perceived as concave; otherwise, the rectangle should be convex [73,89,90,91,92,93,94,95,96,97,98]. The convexity/concavity of the rectangle is determined by whether the shadow contour bends away from the casting edge or toward it. The assumption or Bayes’ prior [99,100] that a single light source comes on the right side from above disambiguates the qualitative shape of the rectangles to be convex or concave.

Despite this explanation, what is puzzling within these two figures is the fact that the rectangles show a clear volumetric effect and, at the same time, they are transparent, empty, or outlined only. Since the volumetric effect is assumed on the basis of the light source, the presence of outline only rectangles contradicts the Bayesian prior. Alternately, it can be suggested that the similarity and grouping induced by the contrast polarity precede the assumption of the light source; thus, the contradiction is only apparent since it occurs at a higher level after the shape formation and organization.

In Figure 7e, the white sides of the rectangles, placed on the extreme arms of the cross, highlight the visibility of the cross, which emerges more and more clearly in Figure 7f,g. The similarity/dissimilarity among components weakens the good continuation and highlights the external boundaries of the cross. Again, the role of the contrast polarity dominates the other principles. The cross is now seen distinctly and directly, while in Figure 7, it appears indirectly as the result of a secondary and holistic visual process related to the arrangement of the external boundaries. Moreover, since Figure 7g shows two objects (the cross and the square) like Figure 7b (two rectangles), the question begs, Which is the outcome to be privileged in terms of simplicity/Prägnanz? The answer to this question is per se very difficult if the role of contrast polarity is not appropriately considered. However, by invoking the contrast polarity, the simplicity/Prägnanz fails when the following conditions are examined.

The grouping strength of contrast polarity is better tested in Figure 7h–j, where new figures, invisible in Figure 6, pop up. The contrast polarity now also plays against the regularity and, more importantly, against simplicity/Prägnanz. In Figure 7h, a straight vertical line, a zig-zagged Greek-like pattern, and a square are perceived. Figure 7i shows an irregular grouping difficult to describe both phenomenally and geometrically, as reported by most observers. In Figure 7j, two equal arrowhead-like shapes pointing in opposite directions have been reported.

Given the similarity/dissimilarity and grouping/ungrouping effects of the contrast polarity within the same pattern of stimuli, the amodal completion can be elicited even when the good continuation is expected to be more in favor of the contrast polarity and, thus, without the need for amodal completion (see Figure 7k–l). In Figure 7k, a horizontal rectangle is amodally perceived behind a vertical rectangle made up and segmented by horizontal white contours. This result entails that the white contours belong to the vertical rectangle, although on the basis of the good continuation, they are expected to group with the horizontal amodal rectangle. Figure 7l shows a strong amodal completion of the partially visible components of the horizontal rectangle, placed behind the vertical segmented one. In Figure 7m, only the right white portion of the figure tends to complete amodally behind the black remaining part of the figure.

In Figure 7n, the two upper and lower portions tend to complete amodally behind the horizontal segmented rectangle. Finally, Figure 7o demonstrates the condition, complementary to Figure 7l, where a horizontally segmented rectangle partially occludes a vertical black rectangle amodally completing behind it.

It is worth noting that, in these new conditions of amodal completion, the rectangles are not perceived only in their perimeter or boundaries. They are not transparent but appear filled and opaque; otherwise, there would not have been any amodal completion. Actually, the continuation of the black vertical contours of Figure 7o appear to complete amodally behind the white contours. This implies a new kind of amodal completion without T-junctions but with I-junctions, where the continuation of contours is behind a contour with the same orientation. The two contours, although with the same orientation, are perceived as two different contours. Further and stronger conditions of this new case of amodal completion will be presented in the next sections.

From these results, the contrast polarity manifests both grouping and ungrouping effects likely due to their salience and visibility, due in turn to the largest amplitude of luminance dissimilarity imparted. In other terms, contrast polarity operates by highlighting components that, on the basis of the resulting similarity and dissimilarity, groups and ungroups accordingly.

The phenomenal results of these stimuli clearly demonstrated the domination of the contrast polarity against good continuation, T-junctions, and regularity. Since they are principles closely related to amodal completion, their role in relation to the contrast polarity is worthy of being more deeply examined to better understand amodal completion in itself and to answer our starting question: What are the role of shape formation and perceptual organization in inducing amodal completion?

As a final remark, Figure 7 questions the effectiveness of simplicity/Prägnanz, information load, and Bayes’ inference. Unless we do not consider the contrast polarity as a constraint or as a prior, Bayes’ inference cannot easily explain these conditions. Moreover, our results cast doubts also on Kolmogorov [101] complexity, which is a natural extension of Occam’s razor. It subsumes the principle of the minimum description length, according to which, given stimuli and a choice of different possible outcomes, it pushes to choose the outcome such that the description of the outcome plus the conditional description of the data is as short as possible. Some of our results, as previously demonstrated, do not conform to this general assumption. Moreover, the high number of possible outcomes previously described represents already a clear weakening of the Kolmogorov complexity. Though many or all the figures can contain a shorter description of the data, it seems that only the contrast polarity chooses the final result, without taking into account either Kolmogorov complexity or the other related principles. We suggest that it is exactly the popping out imparted by the reversed contrast that is the main factor responsible for the described phenomena. The salience and visibility, derived by the largest amplitude of luminance dissimilarity imparted by contrast polarity, precedes any holist or likelihood organization due to simplicity/Prägnanz and Bayes’ inference.

The next step is to test the contrast polarity in more classical conditions used to study amodal completion.

### 3.2. Contrast Polarity and Amodal Completion

**Figure 8:**
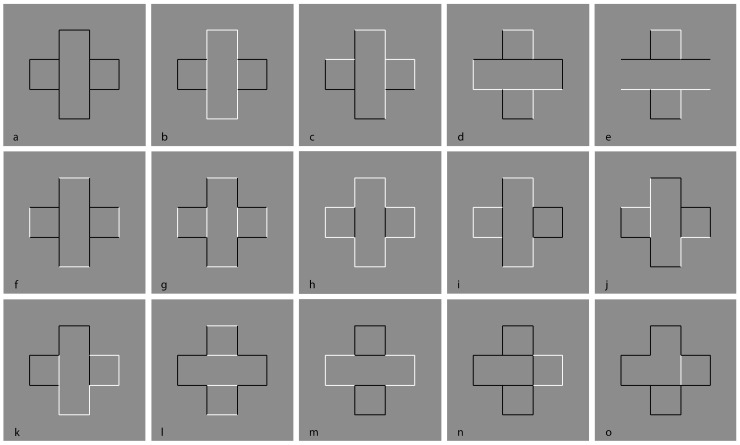
The dominance of the contrast polarity over good continuation, T-junctions, and regularity is also demonstrated within a classical example of amodal completion.

The question of this section is as follows: What is the role of contrast polarity in eliciting amodal completion? The basic stimulus of the following new set of conditions is now shown in Figure 8a, where a classical example of amodal completion is illustrated and spontaneously perceived as a vertical rectangle occluding a horizontal rectangle partially visible on its left and right sides. In Figure 8b, the previous visual organization is strengthened and described as more vivid than the one in Figure 8a.

Figure 8c,d show the volumetric effects, previously described, without the antinomic phenomena induced by the overlapping or intersection of outlined only rectangles. The prior light source from above seems to be effective in both conditions, revealing clear convex (Figure 8c) and convex vs. concave (Figure 8d) results. However, a novel issue resides in Figure 8d, where, despite the T-junctions and the contrast polarity pitted in favor, the amodal completion is absent. Actually, the two upper and lower squares are perceived as bulging closer to the observer than the horizontal concave rectangle. Theoretically, there is not a reason or a constraint in terms of T-junction or border ownership that should prevent or inhibit the amodal completion of bulging objects behind a concave surface. However, there is a physical constraint that should prevent the amodal completion of bulging objects behind a concave surface. It is because, in terms of depth, the concave surface (horizontal rectangle) is further away from the observer than the background and the convex surface (top and bottom squares) is closer to the observer. Therefore, as long as the edges of a convex vertical rectangle and a concave horizontal rectangle are at the same depth as the background, a convex vertical rectangle cannot exist behind a concave horizontal rectangle.

In Figure 8c, two bulging objects are perceived one behind the other. Why should a bulging object not take place behind a concave one? The main point is likely the fact that the bulging squares are perceived closer to the observer than the concave rectangle. This is clearly visible. However, the border ownership and the good continuation show, still clearly, that the side of the square, geometrically in common with the rectangle, is perceived as belonging to the rectangle. As a consequence, the longer sides of the rectangle should be and, indeed, are perceived closer to the observer than the squares.

Furthermore, the squares should be perceived as behind and, since they are incomplete with a missing side, they are therefore expected to complete amodally behind the rectangle. This is not the case. The squares appear in front even if they appear behind the longer sides of the rectangle, which are perceived as behind although the sides are seen in front of the squares. This is a true puzzle or a visual paradox, where different scales and locations of the visual field reveal different and antinomic results that cannot be solved through a unique logic or object hypothesis.

On these bases, both Helmholtz’s likelihood principle and Bayes’ inference are clearly questioned since they use the same global logic in every location and on all size scales of the stimulus. Actually, by removing the two small sides of the rectangle, the antinomy is less strong and the two squares can more easily complete amodally behind the two horizontal contours (Figure 8e). This result is stronger if the two long horizontal sides are not perceived as belonging to the same objects but just as two independent segments. The spatial separation between the two upper and lower parts increases the strength of this outcome.

In Figure 8f, the closure of the components of Figure 8a with white contours improves their figurality and, as a consequence, the amodal completion of the visible left and right elements in a rectangle. In Figure 8g, turning the intersecting contour of Figure 8f from black to white breaks the amodal continuation and induces the pop out of the two lateral squares as complete objects placed in front of the vertical rectangle. Under these conditions, a further case of I-junction, even more salient than those previously described, is perceived. Figure 8h demonstrated that the amodal continuation along the I-junction depends not only on the contrast polarity but also on the arrangement of white and black contours and by the closure principle. The white and black contours segregate in two components, eliciting a white cross with two black contours closing the two horizontal arms but not creating any depth segregation among the regions.

A clear depth segregation is instead visible in Figure 8i, where a vertical rectangle shows, on its left and right sides respectively, an amodal white square-like shape continuing behind its longer side and a complete black square in front of the rectangle. The two lateral shapes reveal a T-junction on the left and an I-junction on the right.

More complex figures to be described are demonstrated in Figure 8j,k, revealing, firstly, an irregular and confusing arrangement of components without any global logic or intelligible likelihood and, secondly, local groupings with local amodal completion or superimpositions separated, independent, and unconnected. Locally, they group and ungroup on the basis of the dominant contrast polarity rule of similarity/dissimilarity.

Figure 8l was described similarly to Figure 8g but with the two squares placed on the upper and lower boundaries of the rectangle and showing again the I-junctions. The effect of the I-junctions is even stronger in Figure 8m, where the white rectangle is clearly perceived as placed and continuing amodally behind and exactly along the sides of the two squares on the top and on the bottom. What is completed is not the surface or area or shape but just the continuous contours of white and black lines.

The last images of Figure 8n,o show conditions that are, again, difficult to describe and locally obey the contrast polarity grouping and ungrouping as previously stated for Figure 8j,k.

### 3.3. Contrast Polarity and Petter’s Effect

**Figure 9:**
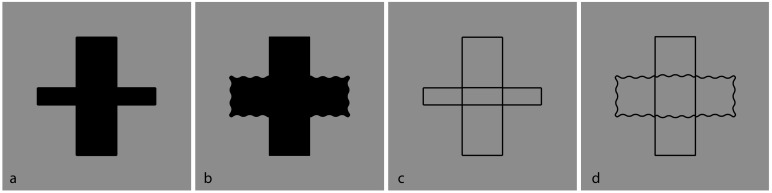
Petter’s effect: the larger surface (**a**) and the region with straight boundaries (**b**) appear in front of the smaller one and of the region with undulated boundaries.

A special case of amodal completion is Petter’s effect [6,86], previously introduced. This effect is seen when a homogeneous black irregular pattern is perceived as made up of independent surfaces separated in depth and delineated by illusory contours in the area of apparent intersection and stratification (Figure 9). Petter [86] discovered that, the larger surface, the region with simpler boundaries and in motion appear, respectively, in front of the smaller, of the one with more complex boundaries, and of the static one.

In Figure 9a,b, two examples of Petter’s effect are shown, where the larger rectangle is perceived in front of the thinner one (Figure 9a) and the vertical rectangle with straight boundaries is seen in front of the one with undulated contours (Figure 9b). Figure 9c,d demonstrates that Petter’s effect can also be perceived with outlined patterns although with less strength.

Petter also suggested a general rule that states that the region with the shorter contours intersecting the one where the surfaces look superimposed has a greater probability of appearing in front of the other surface because it requires a lower energy expenditure. This idea is apparently in accordance with Gestalt theory and with the general “minimum principle” of Prägnanz [45] (Koffka, 1935), suggesting the tendency of neural activity toward minimum work and minimum energy, which spontaneously elicits self-organization toward simplicity. This idea was related to the dynamics of physical systems that naturally converges on a state of minimum energy [19,20,44,79,80,102,103].

Stoner and Albright [104] proposed that Petter’s rule is a consequence of the heuristic and stated that larger figures should be seen in front as a result of perspective projection. Therefore, a close object has a larger retinal projection. Nevertheless, other studies demonstrated that the length of interpolated contours can vary independently from the figure size [105]. These results weaken the connection of minimum principle with likelihood and, ultimately, with simplicity/Prägnanz. The supposed connection can be weakened even more by merely observing Figure 9a–d. The question is why should the solution that is larger in front be simpler than that which is smaller or thinner behind? Moreover, is it more likely that objects with straight boundaries are perceived in front of objects with undulated contours?

These questions become more intriguing by introducing the contrast polarity within the play of Petter’s rule. The main argument is if Petter’s rule is working within a pattern of elements, then it should reduce or amplify the effect of the grouping induced by contrast polarity once the two rules play against the other or synergistically.

**Figure 10:**
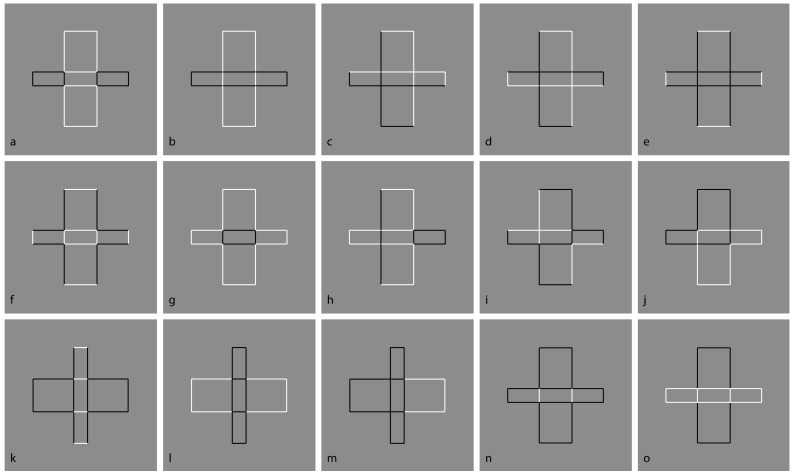
The same set of stimuli of Figure 7 has been redrawn by introducing Petter’s effect according to which the larger rectangle is expected to be seen in front of the thinner one.

To explore this argument, the same set of stimuli illustrated in Figure 7 has been redrawn in Figure 10 by introducing Petter’s effect. The results can be summarized as follows. By comparing each stimulus of Figure 10 with its counterpart of Figure 7, from Figure 10b to Figure 10e, where contrast polarity is in favor of Petter’s effect, the larger rectangle is clearly perceived in front of the thinner one. However, in all the other stimuli, where the contrast polarity breaks the good continuation and the two-rectangles organization, the resulting effects previously described for each stimulus of Figure 7 are in Figure 10 more vivid and stronger. This is likely related to the proximity principle among nearest components, segregated and grouped through the contrast polarity, that favors their emergence as figures segregated from the furthest. For example, in Figure 10a, the two small lateral rectangles are perceived more clearly segregated from the large ones, since they are smaller with nearer sides. Being smaller with close elements elicits a stronger figure–ground segregation as predicted by Rubin’s principles.

There is another, more interesting factor playing in the two counterparts. While in Figure 7a, all the elements are equal in size, in Figure 10a, they are dissimilar in pairs: The two horizontal rectangles differ in size from the vertical ones. This dissimilarity is another factor that favors the amplified effect of segregation in spite of the stronger Petter’s effect that is expected to weaken this segregation. This result suggests that Petter’s effect and, more generally, amodal completion operates after the grouping of the elements occurs on the basis of more primitive and general principles of grouping and figure–ground segregation. The same kind of argument can be used against the global results expected on the basis of simplicity/Prägnanz, unconscious inference, and Bayes’ inference. All the remaining stimuli of Figure 10 obey to the same rationale.

**Figure 11:**
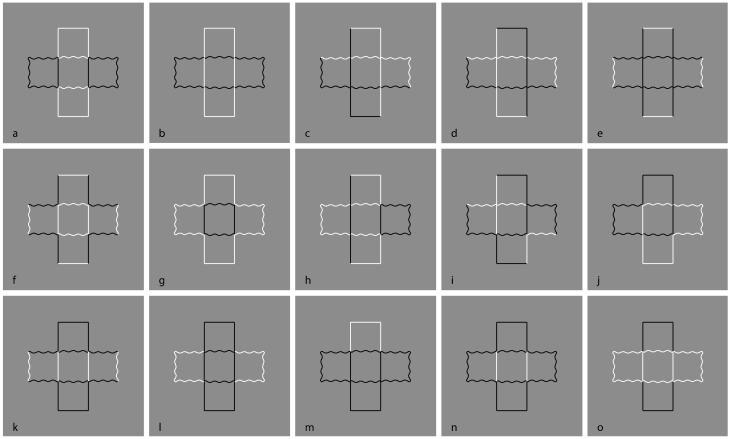
The same set of stimuli of Figure 7 has been redrawn by introducing Petter’s effect according to which the vertical rectangle with straight boundaries is expected to be perceived in front of the one with undulated contours.

In Figure 11, Petter’s figure, as illustrated in Figure 9b, has been extended to the entire set of previous stimuli. In the basic Figure 9b, the similarity/dissimilarity between the two kinds of contours is a further principle useful to test the strength of the contrast polarity. More particularly, in addition to the grouping defined by good continuation and T-junctions, the contour similarity is now used in favor or against the similarity imparted by contrast polarity.

The descriptions of the stimuli were basically the same as their counterpart in Figure 7. The resulting effects, though very effective, were judged slightly weaker in terms of salience and figure–ground segregation.

The only difference between the two sets of stimuli relates to Figure 11d, where the convex–concave effect between the two figures is reversed. This was done to test the same kind of phenomenal attribute, namely, the shape which should be in front is concave and the one which should be seen behind is convex. Therefore, although the two figures are different, their visual meaning is the same.

### 3.4. Contrast Polarity and Tessellation

The figures studied in the previous sections mostly demonstrated the role of contrast polarity in weakening or breaking T-junctions, good continuation, and then amodal completion, both in classical and in Petter’s conditions. There is a limiting case of amodal completion that effectively demonstrates the role of good continuations and of the related junctions. This is the tessellation. We have already seen in Figure 6 potential cases of tessellation made up of adjacent squares. They are like juxtaposed pavement tiles. Phenomenally, this is not the best condition to obtain a true tessellation, since the junctions are such that they let the good continuation flow without changing directions. Therefore, the result is a grid of vertical and horizontal contours, which, in Figure 6, group in two intersecting orthogonal rectangles.

**Figure 12:**
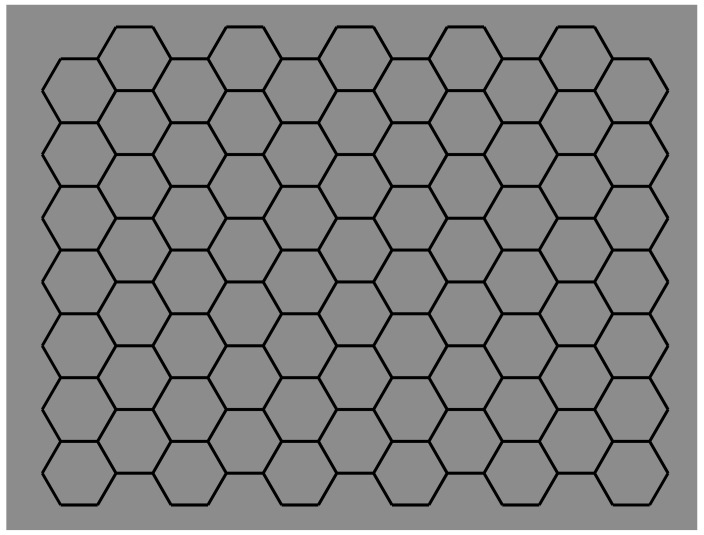
Phenomenal hexagonal tessellation.

A stronger case of tessellation uses hexagons instead of squares, as illustrated in Figure 12. Under these conditions, in terms of the good continuation principle, the bifurcation designed by the Y-junctions elicits two possible directions in perfect equilibrium. Therefore, the flow of good continuation is not stopped by any boundaries responsible for the amodal continuation but proceeds seamlessly. This kind of fluid dynamics description is a metaphorical way to make explicit the role of good continuation with this kind of junction.

Given the grouping/ungrouping effectiveness of contrast polarity, the main question to be answered here is the following: Can contrast polarity elicit amodal completion within a univocal condition of tessellation like the one illustrated in Figure 12?

In Figure 7o, we have seen that reversed contrast switched T-junctions to I-junction. Now, to answer the previous question, contrast polarity is supposed to be able to elicit amodal completion without changing the kind of junctions, i.e., to induce amodal completion with Y-junctions.

**Figure 13:**
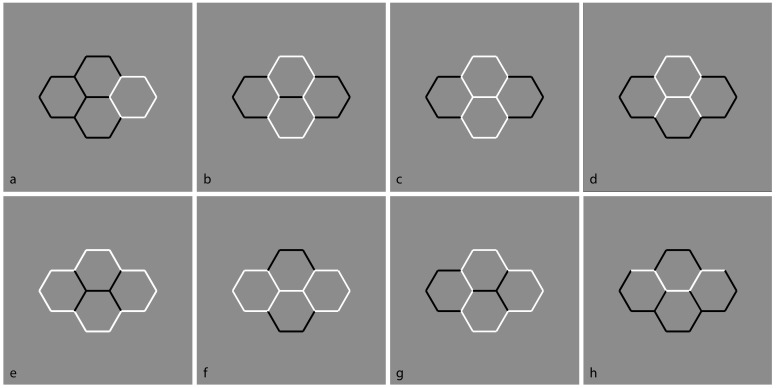
Different kinds of amodal completion induced by contrast polarity within hexagonal tessellations.

In Figure 13, the number of hexagonal cells has been reduced to only four to annul potential global and reference effects of the contest. Figure 13a can be considered the simplest case of amodal completion according to which, by turning one of the four hexagons into a white one, it induces a depth segregation and a change in the inner shape configuration of the two adjacent hexagons that continue amodally behind the white one. Therefore, the oblique sides of the two black hexagons converge amodally and meet in about the center of the white hexagon.

In Figure 13b, the white boundaries become a unique object and, given the unilateral belongingness of the boundaries, induce an amodal completion of the left and right black components that appear like a rectangle-like shape with pointed sides. An analogous amodal result was reported for Figure 13c.

The figure perceived amodally in Figure 13d is a black shape with three pointed sides. In Figure 13e, the white contours are perceived as the boundaries of a four-pointed shape and the inner black elements are its decoration or the visible parts of something larger seen through a window.

In Figure 13f, the upper and lower black contours complete amodally as a kind of rhombic shape behind the two white joined and filled hexagons. Some observers reported something equivalent but in different terms: a diamond face with white glasses.

Figure 13g shows a white shape with three pointed corners in front of some kind of hexagonal shape. It is worth noting that the black contours inside the white shape do not belong to the amodal hexagonal shape but to the white one.

Finally, in Figure 13h, the white contours separating the upper and lower black components are seen as belonging unilaterally to the lower hexagons; therefore, the upper black contours continue amodally behind them.

**Figure 14:**
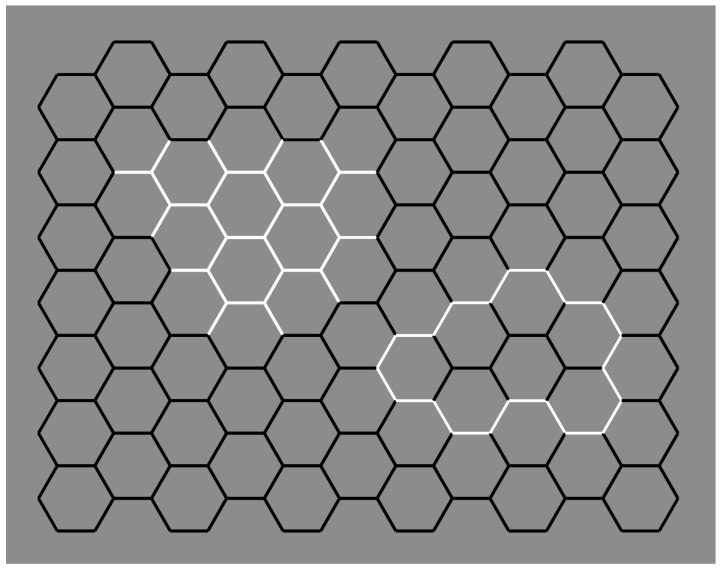
Two different kinds of amodal completion within hexagonal tessellations.

The role of contrast polarity can be expanded and generalized within a larger tessellation (as in Figure 12), thus creating the effective amodal organizations illustrated in Figure 14. Depth segregation and unilateral belongingness of the boundaries are imparted by the contrast polarity in two regions, one bulging closer to the observer and capturing the inner black contours and the other as an irregular window or as a hole showing the white net of contours in depth.

**Figure 15:**
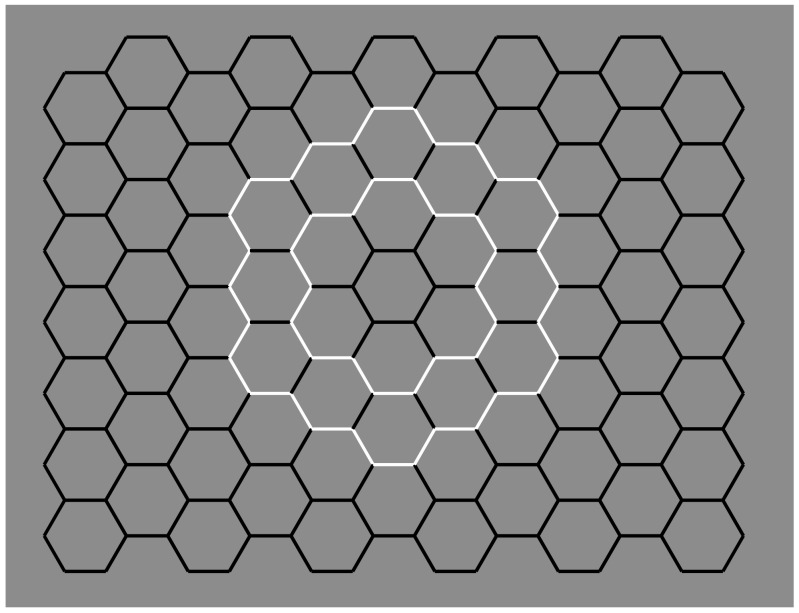
Two polygonal white shapes are perceived as joined together in some kind of annulus or, alternately, two overlapping shapes with the larger behind the smaller one.

In Figure 15, two polygonal white shapes are perceived as joined together in some kind of annulus or alternately as two overlapping shapes, with the larger behind the smaller one. The depth segregation of the surrounding black hexagons is also clearly visible.

**Figure 16:**
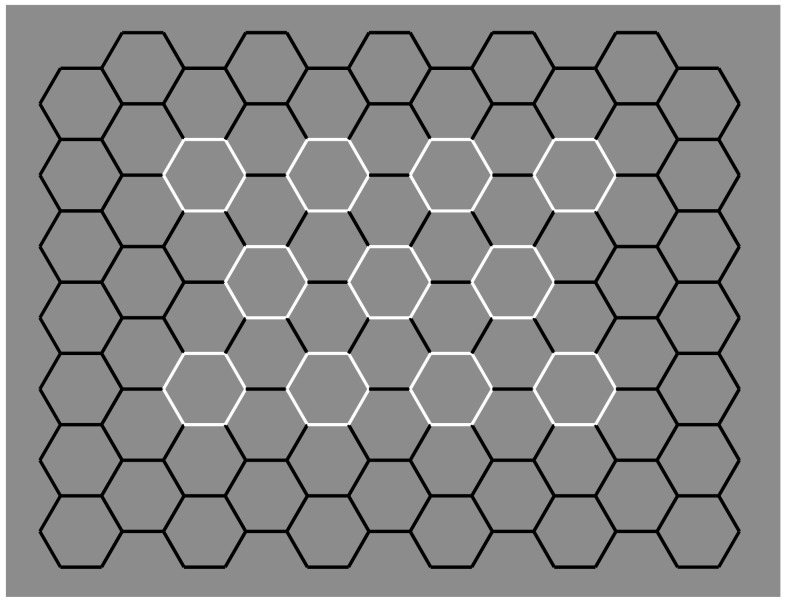
White hexagons partially occluding an amodal texture, different from the surrounding black hexagons.

A further simple but more effective condition is illustrated in Figure 16, where isolated alternated hexagons have been highlighted with white boundaries. Behind them, the amodal perceived texture is very different from the surrounding hexagons, although they should trigger the “unconscious inference” of a homogeneous hexagonal texture according to simplicity and Bayes’ framework.

It is worthwhile to focus on some phenomenal details within this stimulus. First of all, a deformation of the black polygons adjacent to the white ones is perceived according to their amodal continuation behind them. Second, the inner grey of the white hexagons appears different from the one of the black hexagons. This is likely due to contrast-assimilation effects, not further deepened here.

The results of this section support the important role of contrast polarity in perceptual organization. Its strength has been tested here against tessellation, which depends on good continuation and Y-junctions. By taking into consideration all these outcomes, the grouping and ungrouping strengths of contrast polarity could be phenomenally interpreted as a consequence of the border ownership that, in turn, is imparted by the visual salience and highlights effects due to the largest contrast among elements and, thus, by the similarity/dissimilarity among white and black elements. In terms of visual salience, the emergence of the components highlighted through the contrast polarity is, indeed, much stronger than the emergence due to the good continuation alone with its junctions. We suggest that it is exactly the popping out imparted by the reversed contrast that is mainly responsible for the described phenomena. Its salience effect is so strong it dominate other principles when it is pitted against them.

While these approaches are supposed to operate more appropriately at a global and holistic level of vision, explaining the whole percept beyond the single local components, the contrast polarity is a property of the stimulus and a visual attribute that operates locally, eliciting results that could be independent from any global scale and even paradoxical, with contradictory object hypothesis or without any likelihood, as shown in Figure 8 and as reported in the next section. The local highlighted elements of the contrast polarity also manifest global effects when they group together on the basis of the similarity/dissimilarity principle that segregates black and white components as belonging to different visual objects.

### 3.5. Contrast Polarity and I-Junctions

In this section, the I-junctions and the contrast polarity will be tested in limiting conditions that cannot be otherwise explained with the theories considered here. Before introducing the crucial cases, let us first go back to a known effect studied by Kanizsa [9,10,66].

**Figure 17:**
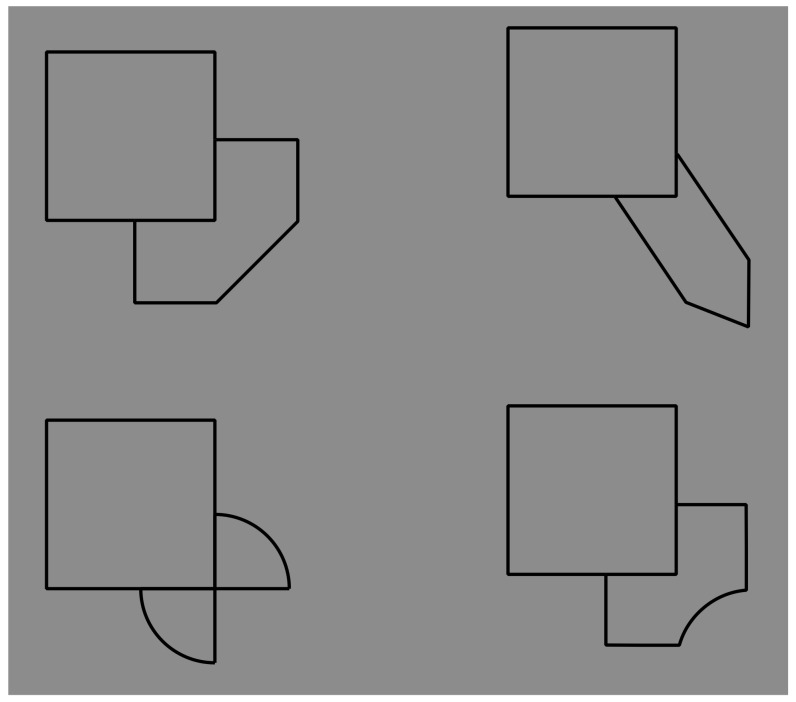
The figures, partially occluded by the square, complete amodally as asymmetrical shapes.

He demonstrated that the shapes of the four partially occluded figures, as illustrated in Figure 17, are not determined by the Gestalt principle of symmetry. In spite of the symmetry and regularity principles suggested by their modal visible portions the amodal shapes perceived are instead influenced by the good continuation of the contours intersecting the occluding square. As a consequence, the amodal completions of the stimuli in Figure 17 appear as four asymmetrical shapes.

**Figure 18:**
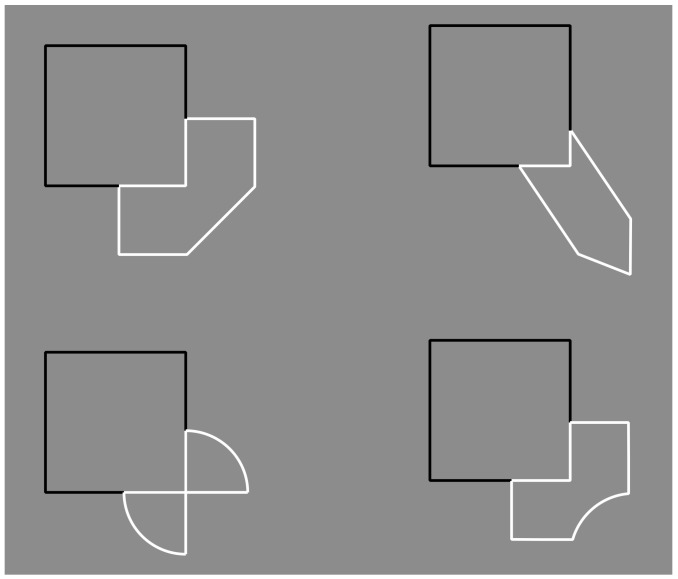
The contrast polarity reverses the amodal completion of Figure 17: The square is now perceived as partially occluded by the asymmetrical objects.

By introducing the contrast polarity as a new player, the occluder–occluded relationship and the amodal completion are reversed (see Figure 18). What previously have been partially hidden by the occluding square now appears as an occluder, and the square in the four conditions is seen partially occluded in its lower right side and exactly on the boundary contours. The I-junction effect is clearly perceived together with the black boundary of the square amodally continuing behind the white boundaries of the white emerging figures. This result occurs also when the luminance contrast among the components is inverted (not illustrated). Again, contrast polarity dominates over good continuation, T-junctions, and regularity.

It could be noted that the third stimulus with two circular sectors with opposed corners due to contrast polarity turns back from irregular, as perceived in Figure 17, to regular in Figure 18. Kanizsa’s argument against regularity and in favor of good continuation is reversed here.

Some might argue that, in Figure 18, the geometrical conditions are restored against the phenomenal ones. This is not totally true since, in Figure 18, amodal completion is still occurring but it is simply reversed in relation to the one perceived in Figure 17.

Within the likelihood and the avoidance-of-coincidences principles, these results are not expected, since the visual system is supposed to prevent interpretations elicited by coincidences. In all the critical conditions studied in this work, the coincidences are not discharged but highlighted.

**Figure 19:**
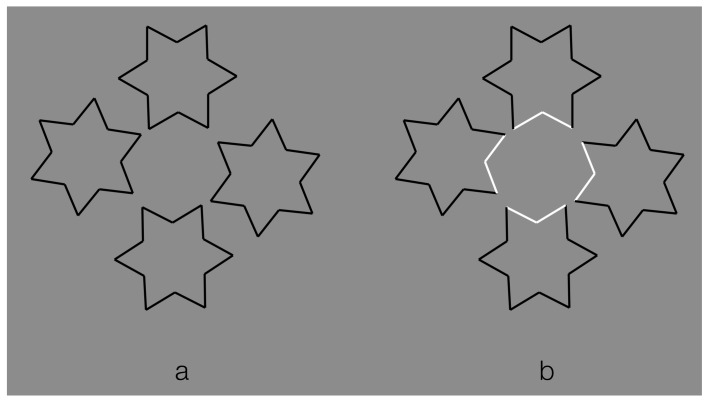
From four stars to a cross partially occluded by a white square-like shape.

The next step is to extend the I-junction effect to conditions where there are no junctions at all but just isolated close figures, like the four stars arranged to create a whole cross, as shown in Figure 19a. It should be noted that the perceived cross is just virtual, namely, a simple arrangement of figures. As a matter of fact, there is not any modal or amodal completion that can combine and unite the four stars that are perceived as such. Figure 19b shows how to join them by reversing the contrast of the four close sides near the center of the group of stars.

Under these conditions, they are not stars anymore but, due to their amodal completion behind the white square shapes, they become a unity, a unique object more similar to a cross (not virtual) with its extreme parts similar to stars.

The following final step is a further extension of our rationale to a limiting case made up of a single shape, i.e., a closed contour, which, by definition, univocally and trivially should be assumed as a unique indivisible object or, at least, made up of sides and angles without any amodal completion that implicitly and explicitly requires two objects placed at different depths.

### 3.6. The Limiting Case of Amodal Completion

As our analysis proceeds, we demonstrate that the strength of the reversed contrast is so great in defining visual objects and amodal completion that it can be considered the optimal tool to test the appropriateness of the theories of which the main purpose is to answer the fundamental question “What is a perceptual object?”.

Since amodal completion can be inverted in conditions where multiple elements play together to create different objects, the question is now the following: Can amodal completion be instilled along the boundary contours of a single shape?

This is indeed the limiting case useful to explore the perceptual principles involved in amodal completion and to test the maximum effectiveness of the contrast polarity. The paradigm of the limiting conditions is reminiscent of the one used in physics to explore the properties of elementary particles, and it is opposite to the holism embraced by Gestalt psychologists [45], mainly based on the assumption that the whole is greater than the sum of its parts, thus implying that the whole cannot be broken down.

**Figure 20:**
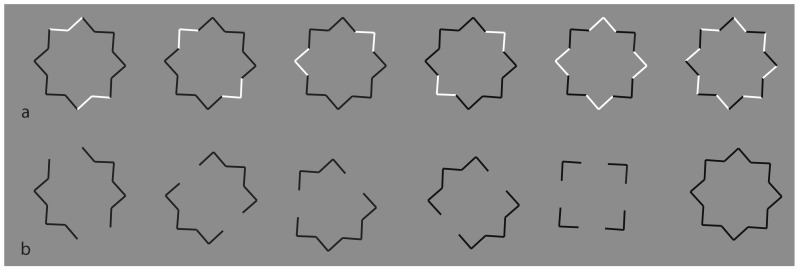
Contrast polarity breaks the unitariness of the stars and elicits amodal completion without junctions (cfr. (**a**) and (**b**) rows).

Following this paradigm, a detrimental and disproving limiting case for both the simplicity and likelihood principles are the conditions illustrated in Figure 20, where the black boundaries of a regular eight-pointed star have been discontinued by reversing the contrast polarity in different portions of each figure. In terms of grouping, the closure principle is mainly responsible for the perception of the eight-pointed star. Also, the good continuation plays a role. Since there are no junctions, the flow of continuation is always good without bifurcations to be chosen or barriers to be amodally completed or continued.

Phenomenally, the uniqueness and wholeness of each star illustrated appear weakened or broken down. Each shape of the first row partially splits into two black and white adjacent components that are seen as belonging to two different things. The two things do not show a traditional amodal completion but something that is anyway comparable to it. In fact, the most extensive black components more easily reveal the formation of a whole shape that, given the segregated white sides become “something else”, do not appear as eight-pointed stars but as the shapes illustrated in Figure 20b. These shapes, shown here as fragments, tend to complete amodally behind the white sides. Although the removal of the white components improves the virtual grouping of the black segments, they appear more clearly as complete and full objects only in Figure 20a, where the closure and good continuation principles are fully functional. The missing sides of Figure 20a do not affect the whole shape but appear as gaps favoring the good continuation of the modal sides and the amodal wholeness [7,68,69,70]. Given the black and white alternation of the sides on the last condition of the first row, the last condition of the first row appears analogous to the star of the last condition of the second row.

**Figure 21:**
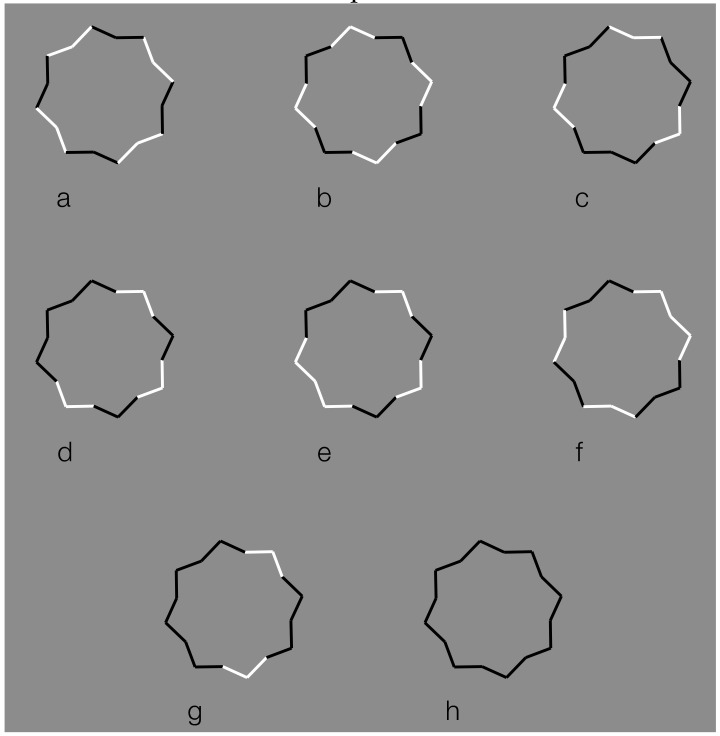
Contrast polarity breaks the unitariness of the stars and induces amodal completion without junctions.

Further examples are illustrated in Figure 21, where the reverse contrast breaks the oneness and the unitariness of the eight-pointed star and significantly influences its shape. New organizations emerge as follows: a concave polygonal shape rather than a star (Figure 21a); two rotated and perpendicular intersecting square shapes with illusory curved sides (Figure 21b); and irregular shapes different from the stars in Figure 21c–g. Figure 21h is the control.

Figure 21a,b displays something that is worth noticing, namely the emergence of the concavity and of the convexity within the star. As a matter of fact, the two figures could be perceived as stars. However, the first appeared as a sort of “eight-concave star” or “eight-indented star” while the second was more similar to a convex star when the subjects were forced to perceive a star. The first, more vivid, and immediate outcome is the one reported above.

The breaking of the uniqueness and unitariness is very effective even though good continuation, closure, and even Prägnanz together favor the grouping. The dissimilarity imparted by the reversed contrast splits the figure. The question is why are these irregular and parceled solutions preferred to a simpler result like “a star with black and white edges”? With this solution, both the whole shape and the local changes would have been preserved and saved. Even if this ideal solution is possible, it does not prevail.

### 3.7. The Limiting Case of the Limiting Case of Amodal Completion

The limiting case of amodal completion is very useful to make explicit the inner phenomenal dynamics. First of all, the contrast polarity can be considered like a barrier analogous but stronger than the one created by T-junctions. Most of our stimuli demonstrated this statement. This barrier is set up by the highest luminance contrast between the black and white poles on the gray background. This is the immediate corollary. This barrier is like a high step phenomenally stating “here starts something different, a new thing.”

The result is the phenomenal salience of something highlighted or accentuated. As a matter of fact, the white elements placed on a gray background pop up with the greatest emphasis since they play with black elements placed on the other extreme of the contrast pole.

It follows that similar highlighted elements can group together on the basis of the similarity principle to elicit a new visual object, to which the boundaries unilaterally belonging. In other terms, the emphasized salient elements group or ungroup on the basis of their similarity or dissimilarity with the remaining elements, thus creating an occluder–occluding duality and, more generally, an effective object segregation and figure–ground organization.

**Figure 22:**
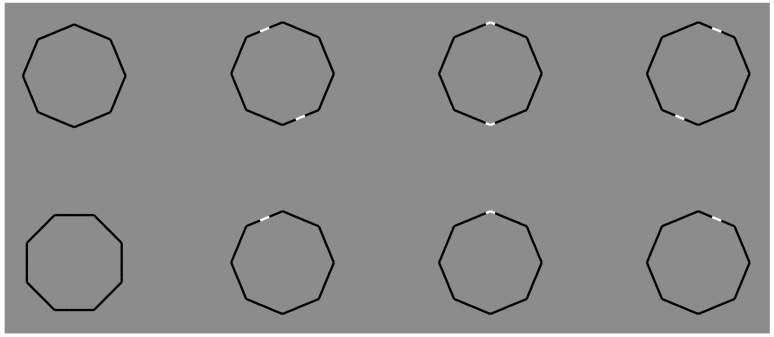
Octagons apparently different due to the accentuation imparted by the contrast polarity.

This is a true sequential dynamics, according to which “the first is the phenomenal salience and, then, the similarity/dissimilarity”. This can be proven through the final set of stimuli illustrated in Figure 22.

In the first column of Figure 22, the two black polygons are the controls: two octagons, on which the main vertical and horizontal axes are placed, respectively, along the corners (top) or on the sides (bottom). They are perceived as two different octagons with accentuated corners or sides. For a full description of these and related figures based on the accentuation principle, see Pinna [11,68,69].

The main predictions can be summarized as follows. The contrast polarity induces a phenomenal salience within the boundaries of the octagon. The salience can become (i) a full object through the similarity principle or (ii) an accent for the attributes of the shape location where the reversed contrast is placed. It becomes a full object if a sufficient number of similar components are present and then grouped. It appears as an accent when few elements or only one play(s) within a large set of elements with opposite contrast.

The creation of a full object has been already demonstrated in Figure 20 and Figure 21. The accent emergence can be tested and seen in the other stimuli of Figure 22, where two (first row) or one (second row) polar accentuation(s) is/are placed on adjacent sides (second and fourth column) or on the top corner of the octagon (third column). All these figures are geometrical replicas of the first octagon on the top resting on one of its corners.

Phenomenally, when the polar accentuations are placed on the sides, the octagons are perceived as tilted (22.5 deg.) polygons, previously resting on one of their sides. They are perceived as more similar to the octagon at the bottom of the first column, although they are geometrically identical to the octagon on the top. Furthermore, they are perceived as tilted in opposite directions in relation to the vertical axis: the first tilted on the left and the second tilted on the right.

When the accentuation is placed on the corners, the octagon is seen like the octagon at the top of the first column. Only one accent (second row) is sufficient to highlight the attributes of the figure where the accent occurs. We called these attributes sidedness and pointedness [58,68,82,106].

Within this figure, we leave out the letters, which are useful to distinguish and name each stimulus, since the letters can also play like accents although less strongly than the contrast polarity [71,107,108].

When the observers were suggested to perceive and report about the segregation or belongingness of the white spots, the most frequent answers were that they can be seen both as part of the polygon or as something different, independent, attached, and extra and as an intruder located above the boundary contours of the polygons.

These outcomes suggest that the white spots behave like true emerging objects placed in front of an amodal black polygon. If this is true, the conditions illustrated in Figure 22 can be considered the limiting case of the limiting case of amodal completion.

## 4. Discussion

In this work, we demonstrated unique and relevant visual properties imparted by contrast polarity in perceptual organization and, more particularly, in eliciting amodal completion that is one of the most common and interesting visual phenomena and compelling issues of vision science. Amodal completion is the vivid completion of a single continuous object of the visible parts of an occluded shape despite portions of its boundary contours not actually being seen. Psychophysical data have demonstrated that T-junction, good continuation, and closure are the main principles involved [1,2,7,14,15,16,17,18].

We discussed the most relevant explanations of amodal completion based on Helmholtz’s likelihood principle [28] and Gregory’s ‘‘unconscious inference’’ [29,30]. According to these theories, the amodal object is similar to a perceptual hypothesis postulated to explain the unlikely gaps within the stimulus pattern and the one that most likely produces the sensory stimulation. Along the same theoretical line, Rock [31,32] proposed the so-called avoidance-of-coincidences principle, stating that the visual system tends to prevent interpretations elicited by coincidences [8,33,34,35,36]. More recently these approaches have been reconsidered in terms of probabilistic Bayesian inference [37,38,39,40,41,42,43], applied successfully in many classical amodal conditions [42,43]. Bayes’ theorem computes a probabilistic decision-making aimed at choosing the amodal outcome as the result of the convolution between the prior p(H), modelling the constraint, and prior assumptions on the structure of the environment necessary to solve underdetermination and the conditional p(D|H) that models optics and the projection on the retina.

Another approach consistent with the previous ones and largely used to explain amodal completion is based on the simplicity–Prägnanz principle of Gestalt psychologists, according to which the visual system is aimed at finding the simplest and most stable organization consistent with the sensory inputs.

All these approaches focus attention mostly on the shape that amodally completes the visible fragments; therefore, they assume amodal completion as the cause of the amodal shape formation. The main interest of this perspective is to explain how the occluded object is completed, what is the amodal shape, and how contours of partially visible fragments are relatable behind an occluder.

Different from these approaches, we adopted the complementary perspective, assuming amodal completion not as the cause but as the resulting effect. The related questions we addressed through our stimuli are the following: “What is the role of shape formation and perceptual organization in inducing amodal completion? What are the perceptual conditions that elicit the segregation of occluded and occluding objects and, finally, amodal completion? What is the role of the local contours, junctions and termination attributes in eliciting the phenomenon of amodal completion?”

Within this perspective, amodal completion has been considered here as a visual phenomenon not as a process, i.e., the final outcome of perceptual processes and grouping principles. Moreover, the contrast polarity with its related similarity/dissimilarity outcomes has been used as the main grouping and ungrouping attribute to explore amodal completion as a visual phenomenon elicited by good continuation, T-junctions, and regularity.

The stimuli were designed as *instantiae crucis* (crucial instances) and studied through the experimental phenomenology, according to the general methods used by Gestalt Psychologists. Together with traditional stimulus configurations, we introduced novel patterns of stimuli, which have been reduced more and more to extreme limiting conditions.

Through our stimuli, contrast polarity has been demonstrated to be effective in inducing amodal completion in conditions where, on the basis of the known principles and of the previous theoretical approaches, it is not expected and vice versa: Amodal completion was annulled or disrupted in the patterns of stimuli where it was supposed to be effective. More in detail, contrast polarity has been able to elicit/disrupt amodal completion when pitted against or in favor of the following conditions reduced to limiting cases: (i) classical patterns (Figure 7 and Figure 8); (ii) Petter’s effect and Petter’s rule (Figure 9, Figure 10 and Figure 11); (iii) tessellation with T-junctions replaced by Y-junctions (Figure 12, Figure 13, Figure 14, Figure 15 and Figure 16); (iv) group of isolated figures arranged in a cross (Figure 17, Figure 18 and Figure 19); and (v) a single shape (Figure 20, Figure 21 and Figure 22).

The results demonstrated the domination of the contrast polarity against good continuation, T-junctions, and regularity. Moreover, the limiting conditions explored revealed a new kind of junction next to the T- and Y-junctions, respectively responsible for amodal completion and tessellation. We called them I-junctions. They elicited the amodal continuation of contours behind a contour with the same orientation.

Contrast polarity was shown to operate locally, eliciting results that could be independent from any global scale and that could also be paradoxical. These results weaken and challenge theoretical approaches based on notions like oneness, unitariness, symmetry, regularity, simplicity, likelihood, priors, constraints, and past knowledge. Therefore, Helmholtz’s likelihood principle, simplicity/Prägnanz, and Bayes’ inference were clearly questioned since they are supposed to operate especially at a global and holistic level of vision.

An alternative explanation of the specific outcomes, particularly related to the limiting conditions of amodal completion, could be based on the phenomenal dynamics made explicit by contrast polarity. First of all, the contrast polarity was perceived like a barrier analogous but stronger than the one created by T-junctions. Most of our stimuli demonstrated this general statement. From this, as a corollary, it follows that this barrier is raised by the highest luminance contrast between the black and white poles on the gray background. This barrier phenomenally represents the starting point for a new emergent “thing”, a new attribute, or a new object.

In other terms, the phenomenal salience, elicited by the highest luminance contrast going from black to white on a gray background, triggers a process of object segregation and its related dynamics: unilateral belongingness of the boundaries and similarity/dissimilarity principles.

The dominance of contrast polarity over good continuation and T-junctions is related to its stronger phenomenal salience and highlight effect. The same argument can account for the emergence of amodal continuation on I-junction, groups of isolated figures, and single shapes. Moreover, the imparted salience can disrupt, both locally and globally, arrangements of figures or can alternately rearrange the elements according to their similarity/dissimilarity. The highlighting strength of contrast polarity determines even the grouping effectiveness against the global and holistic rules and factors expected by Helmholtz’s likelihood principle, simplicity/Prägnanz, and Bayes’ inference.

In favor of the basic and essential role of the phenomenal salience, we can invoke deceiving strategies used in nature by most living organisms. Flowers, birds, and fishes use colors and contrast polarity to attract, reject, show, and hide: to show some parts more clearly than others; to show something that would be otherwise invisible; to show parts that are not natural parts; to show fragments; to show in order to hide; to show not to show; to show to break and split; to show to separate; to show to multiply; to show the oneness; to show some elements, some more or less important elements; to show something and not to show something else; and to show some parts and not to show the whole [85,109]. In short, the phenomenal salience strongly improves the biological fitness of living organisms and, therefore, the capability of an individual of a certain genotype to reproduce and, thus, to propagate an individual’s genes within the genes of the next generation.

The phenomenal salience is a basic requirement also in human beings, in the way we dress, invent fashion and design, in the way we use the maquillage, and in the existence of the maquillage itself [110].

The strength of phenomenal salience imparted by contrast polarity enables the full independence from local or global organizations and top-down or bottom-up dynamics. It eludes all these categories since it can play in favor or against each of them, as demonstrated in our stimuli. It represents a true challenge for the theories discussed here, which cannot easily incorporate it (e.g., as a prior) without losing explanatory power somewhere else. Inside these arguments, it is important to underline that phenomenal salience is a perceptual attribute not restricted to contrast polarity, but it can also be triggered by color, shape, motion and every other visual property [109]. Among them, contrast polarity is one of the most powerful.

Given the significance of this attribute, further experimental studies based on phenomenological, psychophysical, and neurophysiological techniques are required to measure the strength of the phenomenal salience imparted by contrast polarity against other attributes involved in amodal completion. Further studies can shed light on the role of contrast polarity as a general tool useful in testing the range of scientific effectiveness of visual theories, approaches, and models.

Finally, our phenomenological results suggest several extensions and implications for vividness, imagery, and consciousness, shortly described below.

### Implications for Vividness, Imagery, and Consciousness 

There are several phenomenological elements that might turn the scientific attention from amodal completion to the notion of vividness, considered as a property expressing the self-rated degree of richness, details, resolution, and clarity of a mental image, as compared to the experience of actual perception [111] (D’Angiulli & Reeves, 2007). Although this simple definition is supported by a plethora of correlations, studied and measured in literature [112,113,114,115,116,117,118,119,120,121], the function and underlying processes of vividness are still sources of deep scientific challenges and theoretical controversies [111,122,123,124]. Without getting too deep into detail, we suggest that our work might make a contribution by placing new clues and ideas on the debate about the role of vividness in cognitive neuroscience and of its meaning as a phenomenological component of consciousness.

To show how amodal completion could be related to the notion of vividness, we go back to the simple definition of amodal completion reported here: Amodal completion is the vivid outcome of a complete object unity, i.e., the vivid completion as a single continuous object of the visible parts of an occluded shape despite portions of its boundary contours not actually being seen. For our purposes, two interesting terms of this description that are worth highlighting are “vivid” and “completion”. Phenomenally, they belong to close domains although different: “Vivid” is a clear visual outcome under our conditions, and “completion” is in between vision and imagery. This is due to the fact that, even though the sensory experience of completeness and unity is perceptual, the portions of boundary contours actually seen are not directly visible, namely amodal. This distinction becomes more salient under complex, simple, or uncertain conditions (e.g., some of the stimuli shown in the previous sections), where completion elicits different and alternative solutions and mental image formations. Moreover, as a supporting common result, naive subjects spontaneously assume amodal completion as a sort of mental construction (completion) of the invisible part of an object due to past experience, imagery, or some kind of memory association. This kind of ingenuous theory emerges very often at the end of an experiment, testing amodal completion, when subjects ask for its meaning.

Related to the term “completion” is “amodal”, commonly accepted by the scientific community and defined as the vivid experience of completeness without seeing the occluded part of the object. The expression “without seeing” suggests that something that cannot be seen is clearly perceived. This is definitely in between something perceived and something that is not perceived. The need to introduce the term “amodal” is aimed at explaining this uncertain and twofold meanings. Again, the best conditions to perceive the in-between placement of the term “amodal” are specific experimental tasks or ambiguous-complex stimuli (e.g., Figure 6 and Figure 7), requiring time to be clearly seen and described. Under these circumstances, the amodal completion of possible emerging percepts manifests different degree of vividness. 

Indeed, the phenomenological task adopted here can be considered a very functional tool to explore the complexity of these terms, which are, in turn, interesting phenomena to be explored. Moreover, testing simple stimuli, like the previous ones, where grouping principles are pitted one against the other, is useful to explore the no man’s land where vision and imagery meet. Although in everyday life, we perceive effortless complete objects, under unfamiliar, crowded, or camouflaged conditions, the terms “vivid”, “completion”, and “amodal” can easily assume the twofold and in-between meanings described.

A further element is the notion of amodal completion considered as a visual phenomenon, i.e., the final outcome of perceptual processes and grouping principles. This entails that object formation comes first before amodal completion. In short, the priority is to perceive objects, while amodal completion is usually a hidden or a secondary phenomenon. This kind of phenomenal organization clearly improves the biological fitness of living organisms and imparts explicit psychological advantages. Indeed, the need to perceive or create a mental image of an occluded object is compelling since objects are true invariants required in the foreground, while amodal completion can vary widely and can therefore be placed in the background. 

It is not a coincidence that, to perceive amodal completion as a phenomenon, naive people need to be trained; otherwise, it remains in the background or totally invisible. This is also what occurred in the history of vision science. Despite how prevalent and important is this phenomenon, it went unnoticed by scholars for a long time.

More generally, as many other object properties and meanings, amodal completion does not pop up perceptually with the same salience and vividness as others, but it is less prominent and placed at the lower steps of the so-called “gradient of visibility” [59,71].

A final element, even more clearly related to the notion of vividness, is the phenomenal salience, elicited by the reversed contrast. As demonstrated, the contrast polarity can easily play against or in favor of all the objects potentially included in Figure 6, by highlighting one or the other and, conversely, by hiding and making the others invisible. Again, this means that vision always plays under these conditions with modal and amodal perception along the higher and lower steps of the gradient of visibility and consciousness.

All these clues could be useful to explore the role of vividness acting as an index of availability of sensory information and traces and playing a basic phenomenological role in understanding the access to percepts and memories. 

Finally, they suggest that the phenomenal salience imparted by reversed contrast and the gradient of visibility might be, as well as vividness, significant tools to isomorphically define, measure, and express the grades of the conscious experience that pops up from the brain’s complexity of sensory information in perception. In other terms, they express variations and different states of inner first-person experience of the input and the environmental input itself. 

## 5. Conclusions

In conclusion, the vividness of amodal completion under the conditions here studied, together with the phenomenal salience imparted by the reversed contrast and the related gradient of visibility, can be considered phenomenological features of primary sensory consciousness, thus supporting the hypothesis that consciousness is a graded phenomenon.
